# Rectal Etherization

**Published:** 1884-06

**Authors:** 


### RECTAL ETHERIZATION.

This method of administering ether is attracting a great deal of attention just now. In the *Medical Record* of New York, May 3d, we find several articles from surgeons of that city who have tested it in a number of cases. Dr. W. T. Bull, Surgeon to the New York and St. Luke's Hospital, reports seventeen cases. He says:

"The mode of administration was the same in every instance. The ether (Squibb's) was contained in a bottle, which was connected by a rubber-tubing (eighteen inches to two feet in length) with the vaginal nozzle of a Davidson syringe. The tube was introduced into the rectum, and the bottle placed in a vessel containing water at a temperature varying from 120° to 140° F. The ether boiled actively when the bottle was first immersed, and its vapor was given off freely; but at the expiration of five or ten minutes, when the temperature of the water had fallen several degrees, the boiling almost ceased, and it was found necessary to add hotter water to reproduce it Some patients were prepared by withholding food for six or eight hours, and in one or two the bowels were moved by enema before etherization."

"The first "new sensation" experienced has been the distention of the bowel with the gas, but this has not generally been painful, nor given rise to straining. The gas has frequently escaped pretty freely beside the tube At the expiration of three or four minutes the odor of ether has been detected in the breath. The face has then become flushed, the breathing a little slower and deeper, the patients have yawned a few times, and then, when no stage of excitement has ensued, have gradually lost consciousness, breathed stertorously, and all sensation and reflex action have been suspended.

"I have hastened to make public these observations, while they are still too few and too superficial to permit any close study of this method of etherization, because of the one symptom which cannot escape observation, the diarrhœa. Seven out of seventeen patients have had loose passages, containing blood in two instances. In these seven patients the duration of the etherization has varied from ten to forty minutes, and the quantity of ether administered from three to five ounces. There has been little or no pain or tenesmus and no constitutional disturbance accompanying this diarrhœa, which has ceased without the aid of medicine. But its occurrence in so large a proportion of these patients leads me to the conclusion that ether may be very dangerous when employed in this way, and should not be administered recklessly. In even smaller quantities than any of my patients have absorbed it might in young or enfeebled persons produce death from diarrhœa and collapse.

"M. Molliere thinks that anæsthesia by the rectal method is destined to be of great service in many cases. It suppresses the period of excitation; it permits one to regulate the dosage very exactly; it reduces to a minimum the amount of ether needed; it allows the surgeon to operate upon the face; it is a more agreeable method to those patients to whom the odor of ether is nauseating and objectionable. These advantages, which are claimed for the rectal etherization, are not all confirmed by my experience. I find that it does not suppress the period of excitement, and that as a rule a much longer time is required to produce complete anæsthesia than with any of the inhalers or the "towel cone." In several cases it has been impossible to etherize without the aid of the cone. The manipulations are likely to be disagreeable to patients as well as to doctors, and the apparatus cumbersome. It certainly requires *less* ether, and patients are free from the disagreeable odor and the still more disagreeable sense of strangulation; it unquestionably leaves the face free for operations; but it is a dangerous irritant to the intestine.

"In view of these facts I cannot regard the rectal method in any way as a substitute for inhalation, but I shall still consider it a valuable addition to it. To avoid the odor and strangulation one can begin with the rectal administration of a small quantity (℥ ss. to ℨ j.) and then continue with the inhale and in operations on the face this order can be reversed."

Dr. James B. Hunter in the same Journal reports six cases. He concludes his report as follows:

"The method in question promises, in my opinion, to effect a radical improvement in the method of administering ether. A striking feature is the small quantity of ether required, showing how large a quantity is commonly wasted. The absence of any unpleasant sensation on the part of the patient is a matter of no small importance. The rapidity with which anæsthesia can be induced and the general absence of struggling and opposition by the patient give the rectal method a decided value, even if it should be used only as a preliminary to the usual method. It is evidently desirable that the bowels should be freely moved by an enema beforehand."

Dr. Robert F. Weir in the same Journal sounds a note of warning. He says that in the few cases in which he has used it one death has resulted. This was in a child eighteen months old. The child died the morning after the administration of the ether, after having had several large and bloody passages.

The *Record*, commenting on these papers, says:

Our first care, of course, must be to eliminate the possible dangers which may attend such administration Although these can only be practically indicated by extended trial, it is proper that we should discuss in this connection one or two possibilities. Distention of the intestines, although not endangering rupture, may in prolonged operations embarrass the respiration.

"Again it would seem that the degree of etherization may not be always under control. There may be at times, after complete anæsthesia is induced, an amount of vapor in the intestines which may tend to deepen the anæsthesia beyond safe limits. This extra quantity of vapor is practically beyond our reach, as there does not appear to be any means by which we can secure its ready escape.

"In ordinary anæsthesia the cone may be quickly removed in case of danger, the lungs can be allowed to empty themselves, and fresh air can be freely admitted. How an equal degree of safety can be secured for the rectal method under like conditions remains to be proven.

"The number of patients who have subsequently suffered from intestinal irritation is another item to take into account in estimating the relative advantages of the two methods. It is quite possible that these results may be explained by the introduction of condensed vapor, and that improved methods of administration may effectually guard against them.

"It is quite remarkable to note how quickly the vapor is absorbed, some of the patients tasting the ether within two minutes after the introduction of the rectal tube. The general anæsthetic effect is not so rapidly obtained, as a rule, as that by the cone, yet the former administration is less distressing to the patient, and the usual accompaniment of the cone method, the fear of suffocation, is absent. Subsequent vomiting is also less frequent by the rectal than by the old method. Patients recover more quickly from the effects of rectal than from ordinary etherization, and manifest less subsequent excitement. The comparative merits of the two methods are illustrated by the testimony of the patient in St. Francis' Hospital, who had tried both. These are matters which deserve important consideration from many points of view.

"The new method is neither applicable nor necessary in prolonged operations. Even in those upon the face and throat, anæsthesia by the rectal tube need only be alternated with that by the cone, in a large range of cases which will readily suggest itself to the operator. Indeed, in all severe and prolonged operations upon the face a hypodermic injection of eight of ten minims of Magendie's solution of morphine, given a few minutes before cone etherization is commenced, is sufficient, in a large majority of cases, not only to tide over the intervals when the mouth must be free, but to enable the operator to dispense with the ether altogether for varying periods.

"In most nervous persons it may be found advantageous to commence etherization by the rectum until unconsciousness is produced, when the anæsthetic effect can be maintained by the cone with the usual surety and safety. But, as we have already remarked, the range of applicability of the rectal method must be determined by time and experience, and we must be prepared to labor and to wait accordingly."

